# Significant Independent Predictors of Vitamin D Deficiency in Inpatients and Outpatients of a Nephrology Unit

**DOI:** 10.1155/2013/237869

**Published:** 2013-05-12

**Authors:** Recep Bentli, Hulya Taskapan, Halil Toktaş, Ozkan Ulutas, Adnan Ozkahraman, Melda Comert

**Affiliations:** ^1^Internal Medicine Department, Medical Faculty, Inonu University, Malatya, Turkey; ^2^Nephrology Department, Medical Faculty, Inonu University, Malatya, Turkey

## Abstract

*Aims*. Kidney disease was found to be a major risk factor for vitamin D deficiency in a population study of patients hospitalized. The aims of the study were to describe the prevalence of vitamin D deficiency inpatients and outpatients in a nephrology department during fall and to evaluate effect of assessing serum 25-hydroxyvitamin D (25(OH)D) levels and previous supplementation of cholecalciferol on vitamin D status. *Methods*. We studied 280 subjects in total, between October and January. The subjects were recruited from the following two groups: (a) inpatients and (b) outpatients in nephrology unit. We examined previous documentary evidence of vitamin D supplementation of the patients. *Results*. The prevalence of vitamin D deficiency among these 280 patients was 62,1% (174 patients). Fifty-three patients (18.9%) had severe vitamin D deficiency, 121 patients (43.2%) moderate vitamin D deficiency, and 66 patients (23.6%) vitamin D insufficiency. In logistic regression analysis female gender, not having vitamin D supplementation history, low serum albumin, and low blood urea nitrogen levels were significant independent predictors of vitamin D deficiency while no association of vitamin D deficiency with diabetes mellitus, serum creatinine, eGFR, and being hospitalized was found. *Conclusion*. Vitamin D deficiency, seems to be an important problem in both inpatients and outpatients of nephrology. Monitoring serum 25(OH)D concentrations regularly and replacement of vitamin D are important. Women in Turkey are at more risk of deficiency and may therefore need to consume higher doses of vitamin D.

## 1. Introduction

Vitamin D deficiency is reemerging as a major public health problem throughout the world. It is acknowledged that the prevalence of vitamin D deficiency and its associated morbidities are higher than previously thought worldwide. In addition to metabolic bone disease, recent studies reported that vitamin D deficiency could increase the risk of certain cancers, heart disease, and autoimmune diseases including rheumatoid arthritis and multiple sclerosis in adults and diabetes mellitus [1–8]. 

Chronic vitamin D deficiency may have serious adverse consequences in patients with kidney disease [9–13]. To test the hypothesis that low serum 25-hydroxyvitamin D (25(OH)D) levels are a risk factor for kidney disease progression, Melamed et al. analyzed data from 13,328 participants in the National Health and Nutrition Examination Survey (NHANES) III Follow-Up Study, in which 25(OH)D levels were measured from 1988 through 1994, and then participants were followed for up to 12 years. The incidence of end-stage renal disease was 2.6 times greater in people whose serum 25(OH)D was less than 15 ng/mL than in those with higher levels [14]. 

However, it is still not common practice among nephrologists to monitor and correct vitamin D deficiency of patients with kidney disease, because it is widely believed that the capacity of the 1 *α*-hydroxylase to synthesize 1,25(OH)_2_D_3_ decreases progressively because of decreased renal mass and that any vitamin D deficiency associated with calcium-phosphate disturbances is better treated with activated vitamin D. 

Although hypovitaminosis D has been reported frequently in patients with kidney disease, the prevalence of vitamin D deficiency among patients hospitalized in nephrology services is unknown. 

The aims of the study were to describe and compare the prevalence of vitamin D deficiency inpatients and outpatients in the nephrology department during early winter and to evaluate effect of assessing serum 25(OH)D levels and previous supplementation of cholecalciferol on vitamin D status. 

## 2. Material Methods

We undertook a retrospective audit of the serum 25(OH)D levels of inpatients and outpatients in the nephrology departments from October 15, 2010 to January 1, 2011, and previous serum 25(OH)D analysis, and previous vitamin D supplementation history of the patients. The study was approved by the Ethics Committee.

The following information was determined from the patients' medical records: age, gender, primary underlying renal diagnosis, blood urea nitrogen (BUN), serum creatinine, corrected total serum calcium, serum phosphorus, intact parathormone (iPTH), serum albumin levels, recent and preceding results of serum 25(OH)D analysis, and previous documentary evidence of vitamin D supplementation. Estimated GFR (eGFR), using the abbreviated Modification in Diet in Renal Disease (MDRD) equation, was determined [15].

Vitamin D deficiency was defined as 25(OH)D levels below 15 ng/mL (37.4 nM/L). For subanalysis the patients were divided into four diagnostic categories according to their serum 25(OH)D levels: severe vitamin D deficiency (serum 25(OH)D_3_ level, less than 5 ng/mL, (12,5 nM/L)), moderate vitamin D deficiency (serum 25(OH)D_3_ level, 5 to 15 ng/mL (12.5–37.4 nM/L)), vitamin D insufficiency (serum 25(OH)D_3_ level, 15 to 30 ng/mL (12.5–37.4 nM/L)), and adequate vitamin D stores (serum 25(OH)D_3_ level, more than 30 ng/mL (37.4 nM/L)) [16].

## 3. Statistical Analyses

Data are expressed as mean ± SD. The significance of difference between continuous variables was tested using unpaired *t*-test or Mann-Whitney rank sum test and for categorical variables using −2 or Fisher exact test as appropriate. Correlations were tested using Spearman correlation coefficient. *P* values less than 0.05 were regarded as statistically significant. Logistic regression analysis, binary logistic (Hosmer-Lemshow goodness of fit) by enter method, was studied.

## 4. Results

In the local biochemistry database we identified 25(OH)D requests in total 280 inpatients and outpatients in the Nephrology Department between October 15, 2010 and January 1, 2011.

Diagnosis of the patients were given in Table 1. A total of 49 (17.5%) patients were receiving dialysis (17 (34,7%) due to acute renal failure, and all these were inpatients. Sixty-three patients (22.5%) were diabetic. Demographic and clinical characteristics of inpatients and outpatients are shown at Table 2.

The prevalence of vitamin D deficiency among these 280 patients was 62,1% (174 patients). Fifty-three patients (18.9%) had severe vitamin D deficiency, 121 patients (43.2%) moderate vitamin D deficiency, and 66 patients (23.6%) vitamin D insufficiency. Only 40 (14.3%) patients had adequate vitamin D stores. Whereas in patients with vitamin D deficiency, age, BUN, and iPTH were higher, serum albumin, calcium levels were lower. There was no significant difference according to serum phosphorus, creatinine, and eGFR (*P* > 0,05) (Table 3). 

While in 72% of the inpatients (*n*: 90) serum 25(OH)D levels were less than 15 ng/mL (37.4 nM/L), in 54.2% of the outpatients (*n*: 86) serum 25(OH)D levels were less than 15 ng/mL (37.4 nM/L) (*P* < 0.05). While age, BUN, serum creatinine, and phosphorus levels of inpatients were higher, serum 25(OH)D, serum calcium, albumin levels, and eGFR of inpatients were lower than those of outpatients (*P* < 0,05). No significant difference was found according to serum iPTH levels between inpatients and outpatients.

Of 280 patients, 156 (55.7%) had at least one measurement of 25(OH)D levels during the previous followup in our nephrology unit. Of these 280 patients, 55.7% (156) were previously supplemented with cholecalciferol according to vitamin D supplementation protocol of our nephrology unit. The mean duration between recent and preceding measurements of serum 25(OH)D levels and initiation of vitamin D supplementation was 9.9 ± 3.9 months. The preceding mean serum 25(OH)D level was 12.4 ± 6.8 ng/mL (30.9 ± 16.7 nM/L).

Of 174 patients with vitamin D deficiency, 83 (47.7%) and 73 (68.9%) of 106 patients without vitamin D deficiency had vitamin D supplementation history. Of 125 inpatients, 57 (45.6%) and 99 (63.9%) of 155 outpatients had vitamin D supplementation history (*P* < 0,05). No difference was found between patients supplemented with vitamin D previously or not according to BUN, serum albumin, and phosphorus. While inpatients supplemented with vitamin D previously, age (50,8 ± 16,6 versus 58,1 ± 16,7 years), hospitalization days (4,7 ± 9,2 versus 7,9 ± 11,1 days), and eGFR (15,2 ± 21,1 versus 36,5 ± 38,4 mL/min/1.73 m^2^) were significantly lower, and serum 25(OH)D (20,2 ± 18,3 versus 11,9 ± 10,8 ng/mL, 50.4 ± 45.7 versus 29.7 ± 26.9 nM/L), creatinine (7,1 ± 3,7 versus 4,3 ± 3,9 mg/dL), calcium (9,4 ± 3,4 versus 8,7 ± 1,1 mg/dL), and PTH (443,9 ± 468,2 versus 289,6 ± 346,5 pg/mL) levels were significantly higher (*P* < 0.05).

Serum 25(OH)D levels were statistically lower in females than males (15,2 ± 16,8 versus 18,2 ± 14,7 ng/mL; 45.4 ± 42 versus 37.9 ± 41.9 nM/L, resp.) (*P* < 0.05). The prevalence of vitamin D deficiency among these 153 women was 69.3% (106 patients) and 43.5% in men (68 patients) (*P* < 0,05). 

Of 153 female patients with vitamin D deficiency, 91 (56.8%) and 65 (59.3%) of 127 male patients with vitamin D deficiency had vitamin D supplementation history (*P* > 0,05). The mean duration between recent and preceding measurements of 25(OH)D levels and initiation of vitamin D supplementation was 10.3 ± 3.6 months in female patients and 9.7 ± 4.1 months in male patients (*P* > 0.05). No difference was found between males and females according to age, BUN, serum albumin, calcium, phosphorus, PTH, eGFR, and hospitalization days (*P* > 0,05). Serum creatinine was lower in female patients (5,3 ± 3,3 versus 6,6 ± 4,6 mg/dL, resp.) (*P* < 0,05).

In logistic regression analysis female gender, not having vitamin D supplementation history, low serum albumin, and low BUN levels were significant independent predictors of vitamin D deficiency, while we were unable to demonstrate any relationship with vitamin D deficiency and presence of diabetes mellitus, serum creatinine, eGFR, being on hemodialysis, and being hospitalized (Table 4).

## 5. Discussion

Malatya City, which gets lots of sunlight, is at a latitude of 38 : 21 north, longitude 38 : 19 east, and an altitude of 998 m [17]. Average rainfall per year is 384,4 mm. However, 62.1% of our patients had vitamin D deficiency, 23.6%vitamin D insufficiency, while 14.3% of the patients had adequate vitamin D stores. 

Vitamin D deficiency was reported to be high in the general medical inpatient population. The results of a study reported a 57% prevalence of vitamin D deficiency in 290 patients hospitalized in the general medical wards at Massachusetts General Hospital. Sixty-three percent of the patients studied in March and 49% of those studied in September had serum 25(OH)D concentrations of 15 ng/mL (37.4 nM/L) or less. These authors reported that anticonvulsant-drug therapy, renal dialysis, nephrotic syndrome, and winter season were significantly associated with hypovitaminosis D [18]. 

Daoudi et al. [19] from Belgium reported that more than 60% of 332 patients hospitalized presented with serum concentrations below the lower limit of normal (12 ng/mL, 29.9 nM/L), whereas less than 5% reached 30 ng/mL (74.9 nM/L), a level generally recommended to avoid vitamin D insufficiency. These authors reported that young patients are not at a lesser risk of vitamin D deficiency than the older patients and women showed a lesser deficiency than men. 

We found a higher prevalence of vitamin D deficiency in inpatients (63.9%) than those of outpatients (45.6%) in the nephrology department during early winter season (*P* < 0,05). However, in logistic regression analysis being female gender, not having vitamin D replacement previously, low serum albumin, and low BUN levels were significant independent predictors of vitamin D deficiency, while we were unable to demonstrate any relationship with vitamin D deficiency and presence of diabetes mellitus, serum creatinine, eGFR, and being hospitalized.

In our department we follow a vitamin D supplementation protocol which is similar to that recommended by K/DOQI guidelines [16] except cholecalciferol usage. If serum level of 25(OH)D <5 ng/mL (12.5 nM/L), we prescribe 50000 IU of vitamin D3 (cholecalciferol)/week orally/12 weeks and then monthly for six months. If serum level of 25(OH)D is between 5 and 15 ng/mL (12.5–37.4 nM/L), we prescribe 50000 IU of vitamin D_3_ (cholecalciferol)/weekly orally/4 weeks and then monthly for six months.

Of all patients, 55.7% were supplemented with vitamin D previously during followup in our nephrology unit. Vitamin D supplementation history was more common in outpatients. Vitamin D deficiency was less common in patients supplemented with cholecalciferol previously; even vitamin D deficiency was also found among patients supplemented with cholecalciferol during followup. However, we do not have any information on compliance with vitamin D supplements. 

In a retrospective study of 88 patients with CKD stages 1–5 and baseline 25-hydroxyvitamin D level <30 ng/mL (<75 nM/L) performed by Qunibi et al. [20] treatment with ergocalciferol as recommended by K/DOQI guidelines managed to achieve in only 25% > or =30 ng/mL (75 nmol/L). These authors reported that current K/DOQI guidelines are inadequate for correcting vitamin D deficiency in CKD patients and recommended to monitor serum 25(OH)D levels regularly and to give appropriate vitamin D supplementation in order to achieve normal vitamin D status. It seems that our findings are consistent with those of Qunibi et al.

In our study during fall vitamin D deficiency was more common among women (69.3% versus 43.5%); even percentage of the women supplemented with cholecalciferol previously was similar to that of men. In another a cross-sectional study from Turkey Hatun et al. [21] demonstrated that vitamin D insufficiency (43.8%) and deficiency (21%) are common among Turkish adolescent girls at the end of the winter (in April). This finding is more striking in girls who wear concealing clothing, and they did not improve significantly during the summer, whereas the vitamin D status of girls in the other groups did. 

In Turkey, the main source of vitamin D is cutaneous synthesis because there is no food fortification with vitamin D, and supplementation of vitamin D is not a routine practice. Veiling or staying indoors is common in our female patients, although we did not have any information about these in this study. It is possible that these women benefited less from the decreased sunlight through the fall months than men do, possibly by exposing their skin less to the sun. 

Our study has several limitations. Firstly, this study suffers from all the limitations of retrospective observational studies that rely on existent databases, and the information was collected only at one point in time. Secondly, we have no information about compliance with vitamin D supplements. However, we had some information about common practice and results.

As a conclusion, vitamin D deficiency seems to be an important problem in both inpatients and outpatients of nephrology in Turkey. Women are at more risk of deficiency and may therefore need to consume higher doses of the vitamin D. Monitoring serum 25(OH)D levels regularly and replacement of vitamin D are important. A governmental mandate about the supplementation of foods with vitamin D is urgently needed in Turkey. 

## Figures and Tables

**Table 1:** Diagnosis of the patients.

Diagnosis	Frequency (%)
Acute renal failure	13 (4.6)
Chronic kidney disease	230 (82.1)
Acute renal failure on chronic kidney disease	4 (1.4)
Hypernatremia	1 (0.4)
Hyponatremia	1 (0.4)
Glomerulonephritis	8 (2.9)
Renal transplantation	9 (3.2)
Urinary infection	2 (0.7)
Hypertension	12 (4.3)

Total	280 (100.0)

**Table 2:** Demographic and laboratory results of the inpatients and outpatients.

Variable	All patients (*n* = 280)	Inpatients (*n* = 125)	Outpatients (*n* = 155)	*P* value
Age (years)	54.06 ± 16.9 (18–91)	56.9 ± 18 (18–91)	51.8 ± 15.4 (21–84)	0.008
Hospitalization days	—	13.8 ± 11.2 (1–60)	—	—
Serum 25(OH)D levels (ng/mL) (nM/L)	16.5 ± 15.9 (0.4−110) (41.2 ± 39.7)	13.5 ± 15.9 (0.4–110) (33.7 ± 39.7)	18.9 ± 15.6 (1–82.8) (47.2 ± 38.9)	<0.001
Blood urea nitrogen (mg/dL)	54.4 ± 35.54 (7–185)	66.3 ± 37.3 (10–185)	43.1 ± 19.8 (7–102)	<0.001
Serum creatinine (mg/dL)	5.8 ± 3.7 (0.6–17.8)	6.3 ± 3.8 (0.7–17.8)	5.4 ± 3.8 (0.6–16.8)	0.021
Serum albumin (mg/dL)	3.4 ± 0.7 (0.5–4.8)	3.10 ± 0.7 (0.5–4.5)	3.6 ± 0.6 (1.7–4.8)	<0.001
Serum calcium (mg/dL)	9.0 ± 0.9 (6.0–12.7)	8.6 ± 0.9 (6.0–12.0)	9.2 ± 0.8 (7.3–12.7)	<0.001
Serum phosphorus (mg/dL)	4.7 ± 1.6 (1.9–11.9)	5.2 ± 1.8 (1.9–11.9)	4.3 ± 1.3 (1.9–10.1)	<0.001
Serum parathyroid hormone (pg/mL)	376.9 ± 425.9 (10.9–2500)	377.2 ± 362.3 (35.3–2258)	376.8 ± 472.7 (10.9–2500)	NS
eGFR (mL/min/1.73 m^2^)	24.6 ± 31.8 (1.5–125)	16.4 ± 20.8 (1.5–125)	31.3 ± 37.2 (2.1–125)	0.001
Patients previously supplemented with cholecalcipherol		57 (45.6%)	99 (63.9%)	0.003

The data were mean ± SD (min–max) or frequency (%).

**Table 3:** Demographic and laboratory results of the patients with and without vitamin D deficiency.

Variable	Patients with vitamin D deficiency (*n* = 174)	Patients without vitamin D deficiency (*n* = 106)	*P*
Age (years)	55.6 ± 18.1 (18–91)	51.5 ± 14.8 (18–79)	0.033
Hospitalization days	7.8 ± 11.7 (0–60)	3.3 ± 5.9 (0–28)	0.001
Blood urea nitrogen (mg/dL)	59.1 ± 33.8 (9–185)	44.6 ± 25.1 (7–185)	<0.001
Serum creatinine (mg/dL)	5.8 ± 3.8 (0.6–17.9)	5.7 ± 3.9 (0.6–14.7)	NS
Serum albumin (mg/dL)	3.2 ± 0.7 (0.5–4.8)	3.6 ± 0.5 (2.4–4.7)	<0.001
Serum calcium (mg/dL)	8.9 ± 0.9	9.2 ± 0.9 (7–12.7)	0.002
Serum phosphorus (mg/dL)	4.8 ± 1.7 (1.9–11.9)	4.5 ± 1.4 (1.9–8.8)	NS
Serum parathyroid hormone (pg/mL)	398.5 ± 408.3 (20.3–2500)	342.2 ± 452.7 (10.9–2500)	0.004
eGFR (mL/min/1.73 m^2^)	23.4 ± 32.3 (1.5–125)	26.7 ± 31.0 (2.1–125)	NS
Previously treated with cholecalcipherol	83 (47.7%)	73 (68.9)	0.001

The data were mean ± SD (min–max) or frequency (%).

**Table 4:** Logistic regression models for probability of vitamin D deficiency.

Variable	*β*	S.E.	*P*	Odds ratio (95.0% C.I)
Gender (1)	0.907	0.277	**0.001**	**0.404 (0.2–0.6)**
Age	−0.007	0.009	0.404	0.993 (0.9–1.0)
Albumin	0.892	0.253	<0.001	**2.5 (1.5–4.0)**
Diabetes (1)	0.319	0.352	0.365	1.376 (0.7–2.7)
Supplemented with vitamin D (1)	1.188	0.329	<0.001	**0.305 (1.6–0.6)**
eGFR	0.005	0.005	0.309	1.005 (0.9–1.0)
Hospitalization	−0.231	0.322	0.475	1.259 (0.6–2.3)
Put on hemodialysis	0.198	0.409	0.628	1.219 (0.5–2.7)
Constant	−4.488	1.137	<0.001	**0.011**

*n* = 280. Forward stepwise (likelihood ratio) method; entry criteria = *P* < 0.05.

*β*: standard regression coefficient; 25(OH)D, 25-hydroxyvitamin D.
